# The Role of Kv7.2 in Neurodevelopment: Insights and Gaps in Our Understanding

**DOI:** 10.3389/fphys.2020.570588

**Published:** 2020-10-28

**Authors:** Nina Dirkx, Francesco Miceli, Maurizio Taglialatela, Sarah Weckhuysen

**Affiliations:** ^1^Applied and Translational Neurogenomics Group, VIB Center for Molecular Neurology, Vlaams Instituut voor Biotechnologie, Antwerp, Belgium; ^2^Section of Pharmacology, Department of Neuroscience, University of Naples Federico II, Naples, Italy; ^3^Department of Translational Neurosciences, Faculty of Medicine and Health Sciences, University of Antwerp, Antwerp, Belgium; ^4^Department of Neurology, Antwerp University Hospital, Antwerp, Belgium

**Keywords:** KCNQ2, M-current, neurodevelopment, KCNQ2-encephalopathy, Kv7.2

## Abstract

Kv7.2 subunits encoded by the *KCNQ2* gene constitute a critical molecular component of the M-current, a subthreshold voltage-gated potassium current controlling neuronal excitability by dampening repetitive action potential firing. Pathogenic loss-of-function variants in *KCNQ2* have been linked to epilepsy since 1998, and there is ample functional evidence showing that dysfunction of the channel indeed results in neuronal hyperexcitability. The recent description of individuals with severe developmental delay with or without seizures due to pathogenic variants in *KCNQ2* (*KCNQ2*-encephalopathy) reveals that Kv7.2 channels also have an important role in neurodevelopment. Kv7.2 channels are expressed already very early in the developing brain when key developmental processes such as proliferation, differentiation, and synaptogenesis play a crucial role in brain morphogenesis and maturation. In this review, we will discuss the available evidence for a role of Kv7.2 channels in these neurodevelopmental processes, focusing in particular on insights derived from *KCNQ2*-related human phenotypes, from the spatio-temporal expression of Kv7.2 and other Kv7 family member, and from cellular and rodent models, highlighting critical gaps and research strategies to be implemented in the future. Lastly, we propose a model which divides the M-current activity in three different developmental stages, correlating with the cell characteristics during these particular periods in neuronal development, and how this can be linked with *KCNQ2*-related disorders. Understanding these mechanisms can create opportunities for new targeted therapies for *KCNQ2*-encephalopathy.

## Introduction

The *KCNQ* gene subfamily consist of five members (*KCNQ1*–5), all encoding voltage-gated potassium (K^+^) channel subunits (Kv7.1–5). The Kv7.1 subunit is predominantly expressed in the heart tissue, while Kv7.2-5 subunits are expressed most abundantly in the nervous system; Kv7.2 and Kv7.3 subunits expression is mostly restricted to the nervous system, whereas that of Kv7.4 and Kv7.5 subunits are more widespread, also including smooth and skeletal muscles (Sanguinetti et al., 1996; Schroeder et al., 2000; Cooper et al., 2001; Yeung et al., 2007). In adult neurons, Kv7 channels consist most frequently of tetramers containing Kv7.2 and Kv7.3, and more rarely Kv7.5 subunits (Wang et al., 1998; Schroeder et al., 2000). Independently of subunit composition, all Kv7 channels generate voltage-dependent K^+^ currents and represent the molecular basis of the M-current, a voltage-gated and K^+^-selective current which derives its name from its suppression upon activation of M_1_ muscarinic receptors (Brown and Adams, 1980). Muscarinic-dependent M-current suppression is caused by M_1_ receptor-dependent activation of phospholipase C, which hydrolyses phosphatidylinositol-4,5-bisphosphate (PIP_2_), a molecule which is necessary for Kv7 channel opening (Suh and Hille, 2002). The M-current is a slowly activating, non-inactivating, time‐ and voltage-dependent potassium current, which regulates the resting membrane potential (RMP) and dampens repetitive neuronal firing, thereby controlling neuronal excitability (Wang et al., 1998; Brown and Passmore, 2009).

During the last decade, an emerging number of studies pointed out that severe disruption of the function of Kv7.2, Kv7.3, and Kv7.5 subunits due to de pathogenic variants in the encoding gene leads to developmental and epileptic encephalopathy (DEE; Weckhuysen et al., 2012; Lehman et al., 2017; Lauritano et al., 2019). DEEs are a heterogeneous group of mostly neonatal-, infantile‐ or childhood-onset disorders characterized by severe, drug-resistant seizures, characteristic electroencephalographic (EEG) signatures, and different levels of developmental delay or regression, often with a poor prognosis. Although anti-seizure drugs can have some effect on seizure control in *KCNQ*-related DEEs, and several patients become seizure free after a few weeks to months, these patients invariably suffer from severe developmental delay (Pisano et al., 2015). A complex relationship is known to exist between epilepsy and neurodevelopment; before the most recent International League Against Epilepsy classification introducing the DEE term (Fisher et al., 2017), the most-commonly used term of “epileptic encephalopathy” was meant to highlight the classical belief that epileptic activity by itself is the primary cause of cognitive and developmental deterioration. However, some patients can have developmental impairment before seizure onset, or cognitive deterioration may progress despite complete seizure control; given the variable relationship between seizures and cognition, these conditions have been more recently referred to as DEEs. Thus, the possibility that the control exerted over neuronal excitability by Kv7.2, Kv7.3, and Kv7.5 subunits may play a role in neurodevelopment deserves to be investigated. In this review, we will focus on what is currently known about the role of Kv7.2 channels in neurodevelopment.

## Human Pathogenic *KCNQ2* Variants and Neurodevelopment

The best proof for a role of the M-current in neurodevelopment is the observation that pathogenic variants in *KCNQ2* are responsible for a spectrum of neurodevelopmental disorders in humans. Already in 1998, inherited pathogenic variants in *KCNQ2* were identified as a cause of benign familial neonatal epilepsy (BFNE; *KCNQ2*-B; Singh et al., 1998). This disorder is characterized by neonatal seizures, but development in these patients is generally normal (Maljevic and Lerche, 2014). However, the risk of seizure recurrence in BFNE individuals is 10–20 times higher than the general population (Plouin, 1994), and a few BFNE families with individuals affected with various degrees of developmental disability have been described (Dedek et al., 2003; Borgatti et al., 2004). In 2012, some heterozygous pathogenic variants in *KCNQ2* were surprisingly shown to result in DEE, with a developmental delay ranging from mild to profound, nowadays referred to as “*KCNQ2*-encephalopathy” (*KCNQ2*-E; Weckhuysen et al., 2012). Pathogenic variants in *KCNQ2* are shown to be the most common genetic cause of neonatal-onset DEE. A recent prospective cohort study showed that 83% of newborns with a DEE had an identifiable genetic etiology, of whom 40% had a pathogenic variant in *KCNQ2* (Shellhaas et al., 2017). The incidence of *KCNQ2*-E is reported to be 1–3/100.000 births (Lopez-Rivera et al., 2020). By contrast, only a handful of DEE patients with pathogenic variants in *KCNQ3* or *KCNQ5* have been described (Rauch et al., 2012; Epi4k Consortium et al., 2013; Lehman et al., 2017; Ambrosino et al., 2018; Kothur et al., 2018; Lauritano et al., 2019; Rosti et al., 2019; Sands et al., 2019).

**Figure 1:**
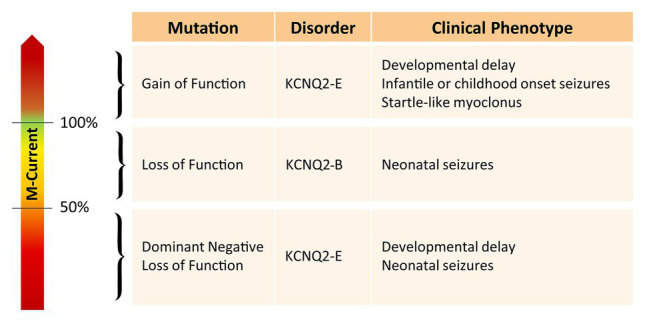
Overview of the effect of different KCNQ2 pathogenic variants on the M-current and their associated clinical phenotype.

Interestingly, the degree of developmental delay does not completely correlate with the frequency or severity of the epileptic seizures (Weckhuysen et al., 2013). Furthermore, there seems to be a clear distinction between pathogenic variants leading to either *KCNQ2*-B or *KCNQ2*-E (Figure 1). Pathogenic variants associated with *KCNQ2*-B are truncating (splice, nonsense, and frameshift), whole gene deletions, or heterozygous missense variants resulting in haploinsufficiency with a 20–30% reduction of the M-current density when mutant subunits are expressed together with wild-type subunits in heterologous cell systems (Maljevic and Lerche, 2014). *KCNQ2*-E, on the other hand, is caused by heterozygous missense or in-frame indel mutations shown to have a dominant negative (DN; >50% reduction of the M-current density), or more rarely gain of function effect (GOF; >100% of the M-current density) when co-expressed with wild-type subunits (Miceli et al., 2013, 2015). Interestingly, *KCNQ2* GOF variants can lead to distinct neurodevelopmental phenotypes. For example, patients with the mild GOF variant R198Q present infantile spasms without prior neonatal seizure (Millichap et al., 2017), whereas patients with the strong GOF variants R201C/H showed severe neonatal-onset encephalopathy without neonatal seizures but prominent startle-like myoclonus and a burst-suppression EEG pattern (Mulkey et al., 2017). *KCNQ2* has also gained interest in the field of autism spectrum disorder (ASD). First of all, many of the reported *KCNQ2*-E patients show autistic features or have ASD as a behavioral comorbidity (Milh et al., 2013; Millichap et al., 2016). Second, *KCNQ2* variants are also commonly identified in genetic studies on ASD cohorts (Jiang et al., 2013; Long et al., 2019). The mechanism by which two opposite effects on the M-channel, either a reduction of more than 50% of the M-current (DN effect) or an increase of the M-current density (GOF effect), both lead to developmental delay, is not known yet. However, this shows that the level of the M-current has to be regulated in a very precise manner to maintain homeostasis of the neuronal network.

Heterologous cellular systems have been used abundantly to study the functional consequences of human pathogenic *KCNQ2* variants, and electrophysiological assays have been used as a read-out to assess genotype-phenotype correlations (Orhan et al., 2014; Abidi et al., 2015; Miceli et al., 2015; Millichap et al., 2017; Kim et al., 2018; Soldovieri et al., 2019). These systems have the advantage of being relatively straightforward, cost-efficient, and relatively high-throughput (Maljevic et al., 2017). The disadvantage of this approach is that the lack of native-neuronal environment may influence channel behavior in such a way that those variant-induced functional changes that affect channel subcellular localization or interaction with neuronally-expressed proteins may not become apparent. This was demonstrated by two recent studies on two different recurrent *KCNQ2*-E variants, A294V and R560W. Both generated similar currents as seen for *KCNQ2*-B variants when overexpressed in heterologous systems (Orhan et al., 2014; Abidi et al., 2015). The DN effect could only be observed in transfected neurons; the A294V variant altered distribution of Kv7.2 containing channels to the somato-dendritic compartment, while the R560W variant reduced Kv7.2 axonal surface expression and impaired gating regulation by phosphoinositides (Kim et al., 2018). This data underline the importance of a neuronal environment to fully understand the disease pathomechanism(s) triggered by each pathogenic *KCNQ2* variant.

An interesting side note is that the Kv7.2 channel has several accessory proteins, many of which are also associated with neurodevelopmental disorders. *SCN1B* encodes for Na_V_β1, a multifunctional molecule that is involved in modulation of sodium and potassium channels (including Kv7.2 channels), neurite outgrowth, axon pathfinding, and cell migration (Brackenbury and Isom, 2011; Nguyen et al., 2012). Bi-allelic pathogenic variants in the *SCN1B* gene are linked with DEE with phenotypic properties similar to Dravet syndrome, whereas heterozygous pathogenic variants lead to generalized epilepsy with febrile seizures plus (Scheffer et al., 2007; Aeby et al., 2019). Another important accessory protein of the Kv7.2 channel is Ankyrin-G, which is encoded by the *ANK3* gene. Ankyrin-G has the essential role to anchor the Kv7.2 subunit at the axon initial segment (AIS) and nodes of Ranvier (Devaux et al., 2004; Pan et al., 2006). Heterozygous pathogenic missense variants have been linked to intellectual disability, ASD, bipolar disorder, and schizophrenia (Ferreira et al., 2008; Bi et al., 2012; Iqbal et al., 2013). Lastly, syntaxin-1A is a member of the soluble N-ethylmaleimide-sensitive fusion protein attachment protein receptors (SNARE) superfamily. The SNARE protein complex facilitates the docking and fusion of synaptic vesicles at presynaptic membrane to enable neurotransmission (Rizo, 2018). Syntaxin-1A can bind to the C-terminus of Kv7.2 subunit, thereby decreasing open probability *via* facilitation of the interaction between the C-terminus and the N-terminus (Regev et al., 2009). To date, malfunction of syntaxin-1A itself is not known to be directly associated with neurodevelopmental disorders, however, pathogenic variants in the gene that encodes for the binding partner syntaxin binding protein 1 (STXBP1) are known to cause several DEE forms including a similar phenotype to *KCNQ2*-encephalopathy (Stamberger et al., 2016). STXBP1-Loss-of-function (LOF) mutations results in an increased binding of syntaxin-1A to the Kv7.2 channel, and thus, a decrease in M-current density (Devaux et al., 2017). Therefore, alterations in M-current density could be a convergence point between STXBP1-E and *KCNQ2*-E.

## Spatiotemporal Expression of Kv7.2 Subunit and its Relation to Neurodevelopment

In this section, we will focus on Kv7.2 channel expression in the central nervous system. However, it should be noted that Kv7 channels are also expressed in the peripheral nervous system (PNS) (Passmore et al., 2003). To date, only few studies are available regarding the Kv7 subunit distribution in the PNS; nonetheless, most of those document a high Kv7.2 and Kv7.3 subunits expression in rat small-diameter dorsal root ganglionic neurons and non-myelinated C fibers (Passmore et al., 2003; King and Scherer, 2012; Huang et al., 2016). In rat peripheral sciatic nerves, most small‐ and medium-sized axons were shown to express Kv7.2/Kv7.3 heteromers, while in the largest sciatic nerve axons, mostly Kv7.2 homomers are observed (Cooper, 2011).

### Kv7.2 Subunit Expression in Mature Neurons in the CNS

Kv7.2 channels are expressed widely throughout the brain in both excitatory and inhibitory neurons. In the adult human brain, immunohistochemistry shows a high level of Kv7.2 channels in the cortex, and lower levels in the hippocampus (Cooper et al., 2000). Within the neuron, Kv7.2 channels are located at the AIS, nodes of Ranvier, the soma, and presynaptic terminals (Cooper et al., 2001; Cooper, 2011). Depending on the Kv7.2 channel subcellular localization in the neuron, Kv7.2 subunits may play different roles. In fact, at the AIS and nodes of Ranvier, Kv7.2 has an important role in stabilizing the RMP and increasing the steady state availability of the sodium channels (Nav) (Battefeld et al., 2014). At the soma, it is a key player for the inhibition of repetitive action potential firing by attenuating after depolarization, and at presynaptic terminals of neurons, the Kv7.2 channel has been shown to regulate presynaptic neurotransmitter release (Martire et al., 2004; Luisi et al., 2009; Regev et al., 2009; Battefeld et al., 2014).

### Temporal Regulation of KCNQ2 Transcripts and Kv7.2 Subunit Expression During Neurodevelopment

#### KCNQ2 Transcripts Expression During Neurodevelopment

*KCNQ2* transcripts are already significantly expressed at the stem cell stage and during the whole course of neurodevelopment, with different transcripts showing differences in temporal expression. *KCNQ2* has six curated transcripts based on the Reference Sequence (RefSeq) database. Transcript a (NM_172107) is the canonical transcript and counts 17 exons. The two most studied transcripts are transcript c (NM_004518; contains a distinct three prime untranslated region and is missing exon 9 and 12) and d (NM_172108; missing exon 12 and has a shorter exon 13), both generating functional channels (Orhan et al., 2014; Abidi et al., 2015; Ambrosino et al., 2015; Devaux et al., 2017). This suggests that exon 9 and 12 are not essential to form a functional channel. In support, no known *KCNQ2*-E pathogenic variant is located in these exons. Transcript b (NM_172106) and f (NM_001382235), both missing exon 12, are not well studied. Lastly, transcript e (NM_172109) is a shorter *KCNQ2* transcript. It consists of exon 1 until exon 8 and has an alternative extension of exon 8 due to alternative splicing, which results in a frameshift. This shorter *KCNQ2* transcript is expressed most abundantly in the fetal brain and its expression levels decrease during neuronal development. Therefore, we will refer to this transcript as the “short fetal transcript.” The opposite is seen for the longer *KCNQ2* transcripts; they are upregulated during neuronal differentiation and increase with ongoing maturation (Smith et al., 2001); however, the probe used in this study recognizes all transcripts, thus, the specific role of each individual transcript cannot be assessed. Nonetheless, in support of this human brain tissue study, the transcriptome data from the LIBD stem cell browser^1^ show a similar temporal expression pattern for the different *KCNQ2* transcripts in induced pluripotent stem cells (iPSCs) and iPSC-derived neuronal cells. This database shows the expression of all exons over the course of neuronal development, from self-renewal toward neuronal progenitor cells (NPCs) and neurons. Interestingly, the data show that exon 1 until 8 (present in the short fetal transcript) are already significantly expressed during the self-renewal stage, and expression stays relatively stable throughout neuronal development. Exon 10–11 and 13–17, but not exon 9 and 12, show a low expression in the self-renewal and NPC stage, with an increased expression when NPCs differentiate into neurons. Lastly, exon 9 and 12 only show a significant expression in neurons. Because transcript c is the only curated long transcript that is missing exon 9 and 12, this suggests that transcript c is the longer transcript seen in self-renewal and NPC stage. Indeed, the expression of the short fetal transcript and transcript c in iPSCs and embryonic stem cells (ESCs) was confirmed in other studies, where they even demonstrated that *KCNQ2* shows the highest expression of all ion channels (Wang et al., 2005; Jiang et al., 2010; Linta et al., 2013). It should, however, be noted that we cannot rule out that additional alternative transcripts without exon 9 and 12 exist that are not included in the curated RefSeq transcripts.

So far, on a protein level, the expression of these different transcript has not been studied during early neurodevelopmental stages. The presence of a shorter transcript of ~40 kDa on western blot has only been observed in adult mouse brain using an antibody targeting the N-terminal of the Kv7.2 subunit (which is conserved among all the Kv7.2 transcripts; Cooper et al., 2001). The size of this transcript is similar to the predicted size of protein encoded by the short fetal transcript. Unfortunately, no subsequent effort has been made in investigating this shorter transcript further.

#### M-Current Density During Neurodevelopment

Functional Kv7.2-containing channels are expressed early in neurodevelopment, and their expression increases during development, thereby influencing neuronal differentiation by altering the neuronal membrane potential. Not with standing the ample evidence at the transcript level for expression of Kv7.2 transcripts c and e from the stem cell stage, evidence at the protein level is so far lacking. In this context, it is however interesting to note that HEK293T cells transfected with the short fetal transcript do not generate a detectable M-current. Co-transfection with the short fetal transcript and the canonical transcript a suppresses the M-current density compared to transfection with transcript a alone (Smith et al., 2001). At the time of this publication, the necessity of PIP2 binding for the opening of the channel was not known yet (Suh and Hille, 2002). Based on the knowledge we have today, the short fetal transcript misses two important PIP2 binding sites, located at the A-B linker and B-C linker (Hernandez et al., 2008; Kim et al., 2018; Zhang et al., 2020). Therefore, if the short fetal Kv7.2 subunit would reach the plasma membrane and would form tetramers, this would result in a strongly reduced open probability of the channel due to reduced PIP2 binding. However, it is very unlikely that the short fetal Kv7.2 subunit will reach the plasma membrane, as it also lacks the subunit-interaction domain (SID), important for tertramerization, and the calmodulin domain, important for the transport to the plasma membrane (Schwake et al., 2003; Cavaretta et al., 2014).

Kv7.2 subunits are nevertheless definitely present at the plasma membrane of immature neurons, and levels are shown to increase during differentiation (Safiulina et al., 2008; Figueiro-Silva et al., 2015; Telezhkin et al., 2018). A low M-current density and a weak Kv7.2 channel expression was shown in neonatal mouse P0 CA3 neurons using a C-terminal antibody recognizing the long transcripts. After the first postnatal weeks, both the M-current density and Kv7.2 channels markedly increase. The low expression of Kv7.2 channels in neonatal neurons allows intrinsic bursting and neuronal synchronization, leading to generation of giant depolarizing potential, which are important for hippocampal network stabilization (Safiulina et al., 2008). Moreover, during neuronal differentiation of iPSC-derived neurons, hyperpolarization of the RMP was shown to be correlated with the expression levels of Kv7.2/3 (Telezhkin et al., 2018). The RMP is a known regulator of cell fate, as highlighted in several studies (Sontheimer et al., 1989; Ng et al., 2010; Rao et al., 2015). To initiate differentiation, mouse embryonic stem cells (mESC) become more depolarized to enter the G1/G0 phase, after which the cell gradually hyperpolarizes during differentiation (Ng et al., 2010). Therefore, more mature differentiated neurons have a more hyperpolarized RMP compared to immature neurons. It is thus not surprising that forced membrane potential changes can alter the fate of cells. (MacFarlane and Sontheimer, 2000; Sundelacruz et al., 2008). This is in line with the observation that overexpression of Kv7.2/3 channels speeded up the maturation of the iPSC-derived neurons and facilitated spontaneous firing, by forcing the RMP to reach the optimal range for Nav channel activation (Telezhkin et al., 2018).

**Figure 2:**
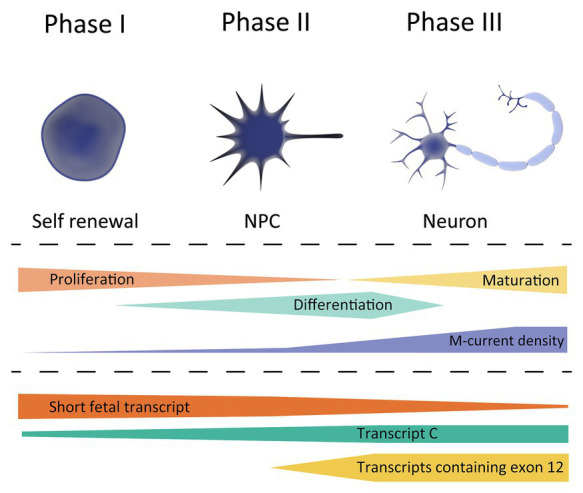
Schematic overview of *KCNQ2* transcript expression during the different phases of neuronal development. Top, showing the different cell types during differentiation; stem cell (left), neuronal progenitor cell (NPC, middle), and neuron (right). Middle, showing the cell characteristics during neuronal development, and its correlation with the M-current density. Bottom, expression profile of the different *KCNQ2* transcripts based on the data exported from the LIBD stem cell browser.

Based on the currently available studies outlined above, we hypothesize that there are three crucial, temporally-distinct phases in M-current density regulated by *KCNQ2* transcript expression that coordinate proper neuronal development. A schematic overview is provided in Figure 2.

**Phase 1**, occurring at very early developmental stages in which a very low M-current density correlates with a strongly depolarized RMP of the cells, a necessary prerequisite to maintain a high proliferation to differentiation ratio. The *KCNQ2* expression profile during this period, i.e., a high expression of short fetal *KCNQ2* and low expression of long *KCNQ2*, makes us believe that the short fetal Kv7.2 isoform serves as guard to prevent a large M-current to be generated and prevent hyperpolarization of the cell. Therefore, it may be hypothesized that pathogenic variants in *KCNQ2* that reduce the M-current will not lead to large clinical consequence during Phase 1.

**Phase 2**, in which an enhanced M-current density occurs as a consequence of a gradual increase in Kv7.2 expression, in order to support neuronal differentiation. A tight regulation of fetal and long Kv7.2 isoforms expression is necessary for a gradual increase in M-current density to hyperpolarize the RMP during differentiation. During the second phase, the first clinical consequence of Kv7.2-mediated developmental channelopathy can be expected.

Finally, **Phase 3**, when a stable M-current density and Kv7.2 expression supports neuronal maturation, corresponding mainly to the postnatal period. During this phase, Kv7.2 channels fulfill their better-known role in neurons; regulating the RMP, dampening repetitive firing, and controlling neurotransmitter release. In this phase, pathogenic variants in *KCNQ2* would lead to the different (mostly-neonatal) phenotypes of *KCNQ2*-B or *KCNQ2*-E, depending on the extent of M-current functional derangement.

#### Temporal Regulation of Kv7.2 and Kv7.3 Subunit Expression

**Figure 3:**
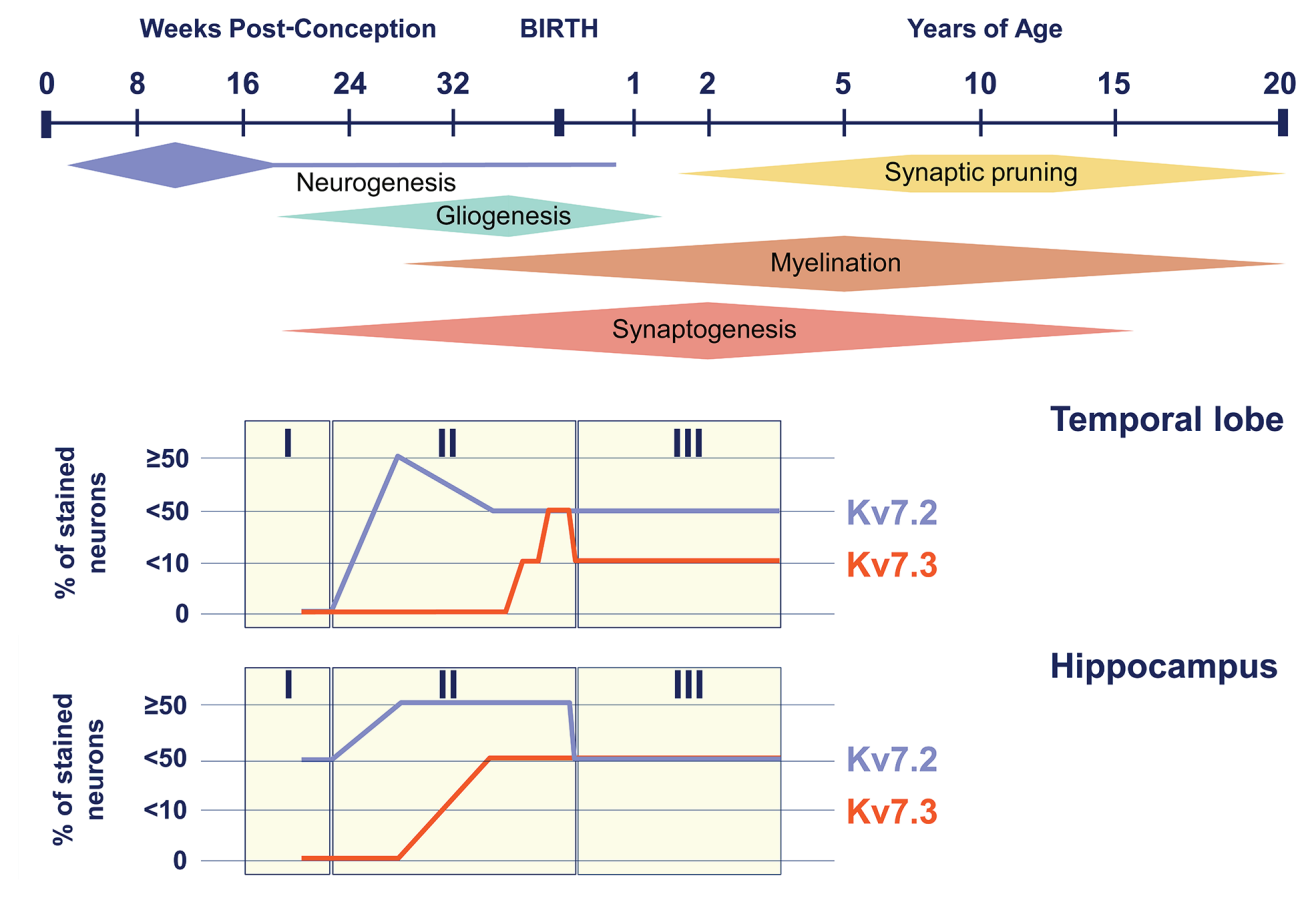
Kv7.2 and Kv7.3 expression during human brain development. Top, timeline of key neural developmental cellular processes in the human brain (adapted from Semple et al., 2013). Bottom, representation of Kv7.2 and Kv7.3 positive neurons in the temporal lobe and the hippocampus during the three neurodevelopmental phases (adapted from Kanaumi et al., 2008).

In addition to the temporal changes in Kv7.2 subunit expression during development, available data also suggest a time-dependent regulation of the expression of other members of the Kv7 subfamily, the Kv7.3 subunit in particular. In mice, Kv7.2 subunits are already significantly expressed at birth, and its levels are relatively stable or even increase postnatally (Tinel et al., 1998; Safiulina et al., 2008). Expression of Kv7.3 subunit, on the other hand, is very low at birth and gradually increases to eventually become even higher than that of Kv7.3 subunit (Tinel et al., 1998; Zhang et al., 2016). Therefore, the ratio of M-channels consisting of Kv7.2 homomers and Kv7.2/Kv7.3 heteromers will shift during development. A similar pattern of age-dependent expression, with Kv7.2 subunits being already detected in fetal life and Kv7.3 subunits increasing from late fetal life to infancy, has also been shown in human brain tissue (Figure 3; Kanaumi et al., 2008). Interestingly, the M-current density of the Kv7.2/Kv7.3 heteromers is 3.8 times bigger than Kv7.2 homomers (Selyanko et al., 2001). Furthermore, when studying DN *KCNQ2*-E variants in heterologous cell systems, the incorporation of the Kv7.3 subunit is able to (partially) rescue the mutational effect on the M-current observed in homomeric Kv7.2 channels (Gomis-Perez et al., 2019). Therefore, the differences in the temporal expression of Kv7.2 and Kv7.3 subunits probably contribute to the typical age-dependent characteristics of *KCNQ2*-related disorders: (i) the neonatal or even prenatal onset, at a time where Kv7.2 homomers are the predominant M-channels and (ii) the transient epileptic phenotype in which the increased expression of Kv7.3 subunits could play a role (Singh et al., 1998; Weckhuysen et al., 2012).

### Kv7.2 Subunit Expression in Fetal Astrocytes

RNA sequencing data show preliminary evidence for early expression of kv7.2 subunits in human astrocytes. In a study on bulk RNA extracted from isolated cell types from mouse and both fetal and adult human cortex, a high expression of *KCNQ2* was found in neurons and oligodendrocyte precursor cells of mice, whereas in humans, the highest expression of *KCNQ2* was found in fetal astrocytes or astrocyte precursor cells, with rather low expression in neurons (Zhang et al., 2016). Open source, adult human brain, single-nuclei RNAseq databases such as the Allen Brain Map^2^ and the UCSC cell browser,^3^ on the other hand, show expression of *KCNQ2* in neurons, but no clear evidence of *KCNQ2* in astrocytes, suggesting expression in astrocytes is restricted to the early neurodevelopmental period. To date, no proof exists for a functional role of Kv7.2 channels in astrocytes. However, if this proves to be the case, this could open a whole new level of complexity to *KCNQ2*-related disorders, as astrocytes are known to contribute to the maintenance and formation of neuronal circuits and are known to play a role in the pathogenesis of several neurodevelopmental disorders, including ASD, Rett syndrome, and Fragile X (Molofsky et al., 2012; Sloan and Barres, 2014; Petrelli et al., 2016)

## Neuronal Cell Models as a Tool to Study the Role of Kv7.2 Subunits in Neurodevelopment

A common method to study the function of M-channels is the use of pharmacological blockers or openers in *in vitro* or *ex vivo* cell models. Retigabine and flupirtine are the most commonly used openers and have a specificity for Kv7.2-5 subunits (Gribkoff, 2003; Yeung et al., 2008). In 2011, retigabine was approved as a treatment for epilepsy by the Food and Drug Administration and the European Medicines Agency; however, it was withdrawn from the market in June 2017 due to side effects including blue discoloration of skin and retina (Tompson et al., 2016). 10,10-bis(pyridin-4-ylmethyl)anthracen-9-one (XE991) and linopirdine are the most commonly used Kv7 channel blockers, both inhibiting all members of the Kv7 family. When interpreting these pharmacological studies, it is, thus, important to realize that none of these drugs are truly Kv7.2 subunit specific.

A clear distinction should also be made between studies using acute and chronic interventions. Acutely blocking the M-current in neurons is known to increase the intrinsic excitability, whereas chronic blocking gradually reduces the intrinsic excitability again. The chronic block induces a compensatory distal shift of the AIS and a relocation away from the soma of Nav and Kv7-channels, thus, again stabilizing network excitability (Lezmy et al., 2017). Chronical blocking of the M-current does not only influence excitability properties but has also been shown to alter neurodevelopmental processes, such as neurite outgrowth and synaptogenesis in different neuronal cell models (an overview of the studies is given in Table 1).

**Table 1:** Overview of *in vitro* studies that use pharmacological blockers or openers to chronically block the M-current.

Cell type	Intervention	Observed effect	References
P0–2 mouse hippocampal CA1 pyramidal neurons	Acute XE991	Increased the intrinsic excitability	(Lezmy et al., 2017)
	Chronic XE991	Gradual decrease in intrinsic excitability and a distal shift of AIS	
PC12 and P0 rat cortical neurons	Chronic XE991	Increased neurite outgrowth	(Zhou et al., 2016)
	Chronic retigabine	Decreased neurite outgrowth	
Rat fetal cortical neurons	Chronic linopiridine	Increased excitatory synaptogenesis	(Figueiro-Silva et al., 2015)
mESC and mouse embryonic hippocampal neurons	Chronic XE991 or chronic linopirdine	Decreased synaptic and vesicular proteins in inhibitory, but not excitatory neurons	(Zhou et al., 2011)

P0–2, 0–2 days postnatally; CA1, cornu ammonis 1; XE991, 10,10-bis(pyridin-4-ylmethyl)anthracen-9-one; AIS, axon initial segment; PC12, pheochromocytoma 12; mESC, mouse embryonic stem cells.

Chronic blocking of the M-current with XE991 in PC12 and P0 rat cortical neurons was shown to result in an increased neurite outgrowth, while retigabine had the opposite effect (Zhou et al., 2016). Additionally, Kv7.2 channels have been described to play a key role in the regulation of synaptic plasticity through its interaction with neuronal pentraxin 1 (NP1) at presynaptic terminals of excitatory synapses and the axonal growth cones of fetal cortical neurons. Chronic blocking of Kv7.2 channels in these neurons increases excitatory synaptogenesis (Figueiro-Silva et al., 2015). Remarkably, another study using chronic XE991 treatment in differentiating mESC and embryonic hippocampal neurons was shown to decrease synaptic and vesicular proteins in inhibitory neurons but not in excitatory neurons (Zhou et al., 2011). A possible explanation for these opposite results could be the difference in studied cell type and cell maturity between the two studies, as Kv7 subunits and transcripts show distinct expression levels, spatial expression, and interactors depending on cell type and maturity stage.

## Mouse Models as Tools to Study the Role of Kv7.2 Subunits in Neurodevelopment

To date, animal models have led to many fundamental discoveries of brain function and have given us a better understanding of the regulation of neurodevelopmental processes. Both knockout (KO) or knock-in (KI) mice models have been generated to study the role of Kv7.2 channels *in vivo*.

### *KCNQ2* KO Mouse Models

The first mouse models to study the function of Kv7.2 channels were KO models, some generated already 2 decades ago. They have taught us that specific neuronal subtypes are more vulnerable to changes in Kv7.2 expression levels and/or function, and especially that loss of Kv7.2 channels leads to complex homeostatic network changes that generally favor hyperexcitability. Heterozygous deletion of *KCNQ2*, engineered in attempt to mimic the human BFNE genotype, fails to prompt spontaneous seizures but lowers the seizure threshold to proconvulsants in adult mice (Watanabe et al., 2000; Yang et al., 2003). The focus of these earlier studies was mainly on seizure susceptibility, as Kv7.2 was not linked yet to behavioral abnormalities. With the current evidence that the Kv7.2 channel plays a role in neurodevelopment and that variants in *KCNQ2* are enriched in ASD cohorts, the *KCNQ2* KO model was also subjected to extensive behavioral analysis; an in-depth behavioral study, indeed, recently showed autism-associated behaviors such as a decrease in social behavior and enhanced repetitive behaviors in *KCNQ2* heterozygous KO mice (Kim et al., 2020).

Homozygous *KCNQ2* KO mice die within hours after birth, which highlights the importance of Kv7.2 channels for survival. To circumvent lethality, brain region-specific *KCNQ2*-null mice have been developed to enable studying complete Kv7.2 channel loss in specific cell types (Soh et al., 2014, 2018; Niday et al., 2017). Transgenic mice with conditional deletion of *KCNQ2* in cortical pyramidal neurons are characterized by abnormal electrocorticogram activity and early death and showed increased excitability of CA1 pyramidal neurons. Interestingly, *KCNQ2*-null neurons did not show alterations in the RMP although they presented a decrease by ~85% of the M-current, suggesting that the contribution of K7.2 subunits to the RMP in p15-p20 mice cerebral cortical pyramidal neurons is small and/or that homeostatic adaptations occur to correct for the absence of Kv7.2 channels (Soh et al., 2014). A follow-up study furthermore showed neuronal hyperexcitability in layer 2/3 pyramidal neurons and alterations in action potential properties (AP), including increase in AP amplitude, decrease in medium after hyperpolarization (mAHP) and a (subsequent) increase in AP frequency (Niday et al., 2017). Specific ablation of Kv7.2 in interneurons, on the other hand, led to elevated excitability of parvalbumin positive (PV+), but not somatostatin positive, neurons. This, in turn, resulted in homeostatic potentiation of excitatory transmission in pyramidal neurons and increased seizure susceptibility in PV-*KCNQ2* null mice (Soh et al., 2018). Importantly, this study shows that Kv7.2 loss induced an increase in interneuron excitability, which influences the course of excitatory network development. In this respect, it is important to point out that γ-aminobutyric acid (GABA) drives excitatory synapse maturation and formation in pyramidal neurons and that early in neurodevelopment, GABA is known to act as an excitatory, instead of an inhibitory neurotransmitter, due to high Na^+^ − K^+^ − Cl^-^ cotransporters 1 (NKCC1) expression (Li and Xu, 2008; Le Magueresse and Monyer, 2013).

### KI Mouse Models of Human Pathogenic *KCNQ2* Variants

Most KI mouse models published so far express human *KCNQ2*-B variants and show a decreased seizure threshold similar to the heterozygous KO mice (Singh et al., 2008; Tomonoh et al., 2014). Interestingly, the BFNE *KCNQ2*-Y284C KI mouse also showed an increase in presynaptic GABA release at brain examination in the first postnatal week (Uchida et al., 2017). Because of the excitatory behavior of GABA early in neurodevelopment, the M-current is one of the most important regulators of neuronal inhibition during this period (Peters et al., 2005). Therefore, the combination of a reduction of the M-current and an increase in GABA release is believed to strongly contribute to the observed neonatal brain hyperexcitability in *KCNQ2*-B.

Until recently, the only genetic *KCNQ2*-E mouse model that had been published was a conditional overexpression model of the DN *KCNQ2*-G279S variant, inserted on the mouse X chromosome (Peters et al., 2005). This variant was modeled based on a DN variant in *KCNQ1*, and has, in the meantime, been described in a patient with *KCNQ2*-E (Sharawat et al., 2019). The mice displayed spontaneous seizures, as well as cognitive and behavioral impairments when scored with the Morris water maze and open-field tests. Morphological characterization of the hippocampus showed loss of mossy fiber terminals in the infrapyramidal layer. Remarkably, the observed phenotype was reversed when mutated *KCNQ2* expression was turned off, using a Tet-off system, in the first postnatal weeks (Peters et al., 2005). In a follow-up study, the authors showed that bumetanide treatment during this same critical period also resulted in a rescue of the phenotype (Marguet et al., 2015). Bumetanide is a NKCC1 antagonist reducing the GABA-mediated depolarization that is seen in early neurodevelopment (Wang and Kriegstein, 2011). In conclusion, both the model with conditional *KCNQ2* KO in PV+ neurons discussed above, the *KCNQ2*-Y284C KI and the *KCNQ2*-E overexpression mouse models, point toward an important role for GABA during early neurodevelopment in *KCNQ2*-related disorders.

Very recently, the first KI mouse model of a recurrent *KCNQ2*-E variant, *KCNQ2*-T274M, has been published, reproducing several phenotypic traits of human *KCNQ2*-E (Weckhuysen et al., 2012; Milh et al., 2020). These mice show learning and memory deficits when tested in the Morris water maze or the Barnes maze, as well as reduced exploratory behavior, suggesting that hippocampal dysfunction is involved in the disease pathology, although no abnormalities were observed upon morphological examination of the hippocampus. Furthermore, these mice present spontaneous clinical seizures starting from P20, which are rarely observed after P100 (Milh et al., 2020). This nicely mimics what is seen in patients, as most of them become seizure free a few months after seizure onset (Weckhuysen et al., 2012). This mouse model will contribute significantly to the understanding of *KCNQ2*-E phenotypic traits, which are often impossible to fully recapitulate in simplistic cell models and will be a very valuable tool for future drug discovery (Maljevic et al., 2017).

## The Role of Kv7.2 Channels in Neurodegeneration

The role of the Kv7.2 channel in neurodegeneration is not the focus of this review; however, it is interesting to note that common genetic variation in *KCNQ2* has recently been associated with risk of cognitive decline in healthy elderly (Bonham et al., 2018). Furthermore, some evidence exists for an (indirect) role of Kv7.2 in neurodegeneration disorders. Amyloid beta (Aβ) oligomers, involved in the pathogenesis of Alzheimer’s disease (AD), were reported to reduce Kv7.2 expression, resulting in a decrease of the M-current (Colom et al., 2011; Leao et al., 2012; Duran-Gonzalez et al., 2013; Mayordomo-Cava et al., 2015). In the medial septal neurons in mice, Aβ seems to reduce the M-current only in the glutamatergic neurons, resulting in increased firing and altered medial septal rhythmicity (Leao et al., 2012). As the medial septal nucleus is a key to generate the theta waves in the hippocampus, which is important for memory formation, alterations in the rhythmicity might underlie the cognitive deficits in AD patients (Winson, 1978; Seager et al., 2002). If the hypothesis is correct that hyperexcitability contributes to cognitive decline, specific Kv7.2 channels openers could be of benefit in AD patients. This could also be part of the explanation why no significant improvements in cognition were observed during a clinical trial of linopirdine, a Kv7.2 blocker, in AD patients (Rockwood et al., 1997). Furthermore, Kv7.2 channels openers could also be beneficial for Huntington’s disease (HD), as retigabine has been reported to rescue the electrophysiological and behavioral phenotype of transgenic HD model (Cao et al., 2015). Moreover, retigabine was described to block the hyperexcitability and to improve motor neuron survival of iPSC-derived motor neurons from amyotrophic lateral sclerosis patients (Wainger et al., 2014). Lastly, for Parkinson’s disease, several studies have shown that blocking of Kv7 channels with XE991 increased excitability of the dopaminergic neurons and has a neuroprotective effect toward dopaminergic degeneration in a mouse model of Parkinson’s disease (Shi et al., 2013; Liu et al., 2018). Altogether, those results support the idea that Kv7 subunits, possibly including Kv7.2 subunits, are interesting therapeutic target for different neurodegenerative diseases and cognitive impairment.

## Further Perspectives

Since the discovery of the *KCNQ2* gene in 1998, *KCNQ2*-encoded Kv7.2 subunits have been studied extensively (Singh et al., 1998). Although multiple lines of evidence show that Kv7.2 channels are involved in neurodevelopment, most studies have focused on the function of the Kv7.2 channels in a mature neuronal environment. Therefore, in this review, we highlight the importance of Kv7.2 in (early) neurodevelopment. Thanks to the increasing use of RNA-sequencing and the availability of transcriptome databases, a large amount of information on *KCNQ2* expression has become available, leading to the consensus that an increasing expression of this gene is consistently observed during neuronal development (Safiulina et al., 2008; Telezhkin et al., 2018). Interestingly, multiple transcriptome datasets show evidence for the expression of a shorter fetal *KCNQ2* transcript (transcript e) in iPSCs and neuronal derivatives, in addition to the longer transcript transcript c (Wang et al., 2005; Jiang et al., 2010; Linta et al., 2013). The presence of the short transcript was already reported in 2001 in brain, both at the transcript and protein levels; however, no follow up studies investigating this transcript were performed (Cooper et al., 2001; Smith et al., 2001). Unfortunately, functional evidence for the presence of Kv7.2 protein at stem cell and neuronal progenitor stages is currently lacking. Future studies confirming or negating the presence of Kv7.2 isoforms in stem cells will be needed to clarify this issue. In the meantime, and based on the available data, we introduce a model dividing the M-current density in three phases: (1) very low density important to maintain cell proliferation, (2) increasing M-current density to hyperpolarize the membrane and support neuronal differentiation, and (3) a stable high M-current density present in maturing or mature neurons to dampen repetitive firing and control neurotransmitter release.

Regarding human disease related to Kv7.2 channel dysfunction, more specifically *KCNQ2*-E, it is clear that pathology goes beyond a simple increased open or closed state of the channel, but includes impact on temporo-spatial expression levels, binding partners, and widespread homeostatic network changes that are difficult to study in simplified models. The recent publication of a *KCNQ2*-E KI mouse model is certainly promising and will be an important tool for further in-depth studies (Milh et al., 2020). Another promising model that would contribute to our understanding of the role of Kv7.2 channels in neurodevelopment is neuronal cultures derived from human iPSCs, as they provide the neuronal environment which is lacking in heterologous cell systems. Their ability to differentiate in several different brain cell types allows us to look not only at network changes but also to the contribution of different cell types to pathology. They are especially useful in the study of neurodevelopmental disorders, since they largely recapitulate fetal development (Tidball and Parent, 2016; Logan et al., 2019). Compared to monolayer neuronal cultures, brain organoids have the ability to foster even more complex cell interactions that stimulate cellular maturation, better recapitulating potential pathological cascades (Logan et al., 2019). Both 2D iPSC-derived neuronal cultures and 3D brain organoids have already successfully been used for the study of several neurodevelopmental disorders to identify disease mechanisms and potential new therapeutic targets (Fink and Levine, 2018; Russo et al., 2019). To date, no iPSC-derived neuronal model for *KCNQ2*-E has been reported in the literature yet. Furthermore, the current research performed to understand the role of the Kv7.2 channel in neurodevelopment has focused mainly on LOF of Kv7.2 channels, whereas *KCNQ2* GOF variants give rise to an at least equally severe neurodevelopmental phenotype in human patients (Miceli et al., 2015). How both DN and GOF variants lead to neurodevelopmental delay is not understood yet. Future efforts comparing the effects of both DN and GOF variants on the neuronal network *in vitro* and *in vivo* are necessary to understand how Kv7.2 regulates neurodevelopment and can potentially open new doors for innovative therapeutic tools. Lastly, the development of Kv7 channel blockers and openers overcoming some of the pharmacokinetic and pharmacodynamic limitations shown by the available drugs (all of which have been synthesized and started to be developed well before the discovery of the *KCNQ* gene subfamily and the description of their multiple pathophysiological roles) could not only be beneficial for patients suffering from *KCNQ2*-B and *KCNQ2*-E but could also positively impact patients affected with multiple neurodegenerative disorders (Wainger et al., 2014; Kumar et al., 2016; Ostacolo et al., 2020).

## Author Contributions

All authors listed have made a substantial, direct and intellectual contribution to the work, and approved it for publication. ND and SW designed the scope and structure of the review. ND, SW, FM, and MT wrote and edited the final manuscript.

### Conflict of Interest

The authors declare that the research was conducted in the absence of any commercial or financial relationships that could be construed as a potential conflict of interest.

## Footnotes

^1^http://stemcell.libd.org/scb/

^2^https://celltypes.brain-map.org/rnaseq/human/cortex

^3^https://autism.cells.ucsc.edu/
